# One-Dimensional CoMoP Nanostructures as Bifunctional Electrodes for Overall Water Splitting

**DOI:** 10.3390/nano12213886

**Published:** 2022-11-03

**Authors:** Xin Chang, Jun Yan, Xinyao Ding, Yaozu Jia, Shijie Li, Mingyi Zhang

**Affiliations:** 1 Key Laboratory for Photonic and Electronic Bandgap Materials, Ministry of Education, School of Physics and Electronic Engineering, Harbin Normal University, Harbin 150025, China; 2 National Engineering Research Center for Marine Aquaculture, Institute of Innovation & Application, Zhejiang Ocean University, Zhoushan 316022, China

**Keywords:** CoMoP, OER, HER, CNFs, electrospinning

## Abstract

As high-quality substitutes for conventional catalysts, the bifunctional catalytic properties of the coating of transition-metal-based materials are pivotal for improving water-splitting efficiency. Herein, cobalt-molybdenum bimetallic phosphide nanofibers (CoMoP NFs) were synthesized via a series of facile strategies, which are divided into pyrolysis electrospun PAN and metal salts, to obtain one-dimensional morphology and a gas-solid phosphating precursor. The obtained CoMoP NFs catalyst has superior catalytic activity performance in 1M KOH. Serving as an oxygen evolution reaction (OER) catalyst, the electrode of the CoMoP NFs affords different kinds of current densities at 50 mA cm^−2^ and 100 mA cm^−2^, with low overpotentials of 362 and 391 mV, respectively. In addition, the hydrogen evolution reaction (HER) performance of the CoMoP NFs mainly shows when under a low overpotential of 126 mV, which can deliver a current density of 10 mA cm^−2^. In order to further detect the stability of the catalyst, we used multiple cyclic voltammetry and chronopotentiometry tests for OERs and HERs, which maintain performance and carry a current density of 10 mA cm^−2^ for longer. As an integrated high-performance bifunctional electrode for overall water splitting, the CoMoP NFs only require 1.75 V@10 mA cm^−2^ for 40 h. This work highlights a facile method to create an electrocatalyst with fiber nanostructures which possesses excellent activity as an alkaline electrolyte.

## 1. Introduction

The most recent popular topics include protecting natural resources and the environment, which human beings depend on for their survival [1,2]. To fulfill the above aim, grant attention has been devoted to simplifying production operations that can produce eco-friendly energy to promote practical utilization. There are many ways to sustainably generate high-energy density resources, such as synthesizing hydrogen. Using electrochemical water-splitting replaces traditional extraction methods, thus reducing environmental harm [3,4,5]. Complex kinetic reactions that occur on the electrode surface during the electrolysis of water, which consists of oxygen evolution reaction (OER) and hydrogen evolution reaction (HER), causing difficulties in reaching the equilibrium potential of the thermodynamic theory of the ideal electrolysis of water (E^0^_RHE_ = 1.23 V and 0 V). Pt- and Ru-based materials, which are state-of-the-art catalysts, are considered to be superior for decreasing the overall energy barriers of water splitting. The utilization of similar precious-metal-based catalysts practically shrinks as a result of high costs, resource scarcity, and unfavorable stability [6,7,8,9].

As a way to solve the above problems, non-noble metal-based electrocatalysts have been widely used by most researchers [10,11,12,13,14]. Transition metal-based carbides, such as sulfides, borides, nitrides, selenides, and phosphides, have been proven to have superior electrocatalytic properties for the production of oxygen or hydrogen [15,16]. Among them, transition-metal-based phosphides (TMPs) show broad prospects in terms of alkaline electrolyte aspects, with excellent stability [16,17,18,19,20,21]. However, the insufficient reactivity of single metal phosphides (e.g., FeP [22], Ni_2_P [23], and CoP [24]) hinder this as an application. The current reports showed that the addition of a second metal to the phosphides could play an effective role in coordinating the metal ions and improving the inherent activity of the electrocatalysts. However, the actual role of the binary metal ions in the electrocatalysts and their associated structural evolution is unclear. Therefore, it is of great significance to synthesize a bifunctional electrocatalyst in a facile way for overall water-splitting efficiency [25,26,27]. Binary TMPs are more inclined to form the electron structures to transform into oxyhydroxide, which, when generated, undergoes electrochemical activation and is converted into OER, which can enhance catalytic performance with the corresponding active materials [28,29]. Apart from the above reasoning, the synergistic effects of different metals can also optimize the efficiency of electrochemical catalysis. As aspects of the HER process, P atoms, as proton receivers, are responsible for the adsorption of H^+^ in the electrolyte, while different metal elements copromote the charge distribution of the P atom, which contributes to the release of hydrogen [30]. At present, the preparation methods of TMPs mainly include (i) solution-phase synthesis, organic tri-phenylphosphine, and tri-octylphosphorus reacting with metals to obtain phosphide; (ii) electrochemical deposition, with phosphides deposited on conductive substrates in the metal salt solution; (iii) thermal decomposition reduction, whereby materials with PO^4−^ groups are used to complex with metal ions in high-temperature reaction systems to prepare metal phosphides; and (iv) the gas-solid reaction, using NaH_2_PO_2_, NH_4_H_2_PO_2_, and P split into PH_3_ gas, which uses phosphating precursors, such as metal oxides and hydroxides, to generate the metal phosphides [31,32,33,34]. Among them, gas-solid reaction technology is a feasible way to obtain the expected catalysts. On the other hand, a suitable structure design for the catalyst can form bigger active areas with electrolyte contact that is beneficial for ions and gas emission. Fiber-like nanostructures are notable because a large space exists in their cross-link network and surfaces, which leads to the fast transportation of the charge. Electrospinning, with the advantage of controllable and efficient operations, is a feasible strategy for modified, ideal fiber morphology, which has tremendous application in various energy production industries. Although sacrificing the stencil of a polymer compound, the resulting materials maintain a uniform appearance, with a nanostructure suitable for extraordinary catalysis capacity toward overall water splitting.

With the aim of embedding a second metal and obtaining a stable-fiber nanostructure, we synthesized novel catalyst CoMoP nanofibers derived from CoMoO_4_ nanofibers (CoMoO_4_ NFs) via simple technologies, including electrospinning and gas-solid reactions. As expected, the CoMoP NFs exhibit breakthrough OER activity at 1 M KOH; when reaching a current density of 50 mA cm^−2^, they only required a low potential of 362 mV. In the same alkaline condition, the CoMoP NFs show excellent performance for hydrogen production, which delivers a current density of 10 mA cm^−2^, only requiring a low overpotential of 126 mV (being kept with a small overpotential after long-term CV tests for the HER process). Significantly, the application of bimetallic phosphide catalysts is a fascinating area of development for enhancing the efficiency of electrocatalysis, with excellent fix activity during CV cycling tests. Indeed, a bifunctional CoMoP NFs catalyst performs overall water splitting at a current density of 10 mA cm^−2^ for 40 h with a cell voltage of 1.75 V.

## 2. Experimental Section

### 2.1. Materials

Cobalt acetate tetrahydrate (C_4_H_6_CoO_4_·4H_2_O), potassium hydroxide (KOH), sodium hypophosphite (NaH_2_PO_2_·H_2_O), phosphomolybdic acid hydrate (H_5_Mo_12_O_41_P), and N, N-dimethylformamide (DMF) were purchased from Zhiyuan Reagent Corporation (Tianjin, China, Alfa Aesar). Polyacrylonitrile (PAN) was bought from Sigma-Aldrich Corporation.

### 2.2. Synthesis of CoMoO_4_ Nanofibers

Preparation of the precursor solution: 149.43 mg C_4_H_6_CoO_4_·4H_2_O, 91.26 mg H_5_Mo_12_O_41_P, and 0.5 g PAN are dissolved in 5 mL of DMF, orderly. Stirring the above solution for almost 10 h constantly forms a pure green solution. Then, we transferred the precursor solution into a syringe, while connecting with a tiny carbon rod. We then performed continuous spraying for 10 h before obtaining a thick round fibrous natural felt, with the voltage set as 7 kV. Then, the PAN/metal salts felt was calcined at 500 °C for 3 h, with a rising heat time of 260 min; the obtained CoMoO_4_ nanofibers are named CoMoO_4_ NFs.

### 2.3. Synthesis of CoMoP Nanofibers

CoMoP nanofibers are prepared as follows: 15 mg CoMoO_4_ NFs and 300 mg NaH_2_PO_2_·H_2_O are set in two separate sides in the railboats, with NaH_2_PO_2_·H_2_O as the P source on the upstream side of tubular furnace. The furnace is calcined at 800 °C for 3 h, with a rising speed of 5 °C min^−1^ for nitrogen flow, while the most important step of the heating process is kept at 250 °C for 30 min during the heating procedure. After the furnace cooled to temperature, the gray product obtained was the CoMoP nanofibers and was signed as CoMoP NFs. For the synthesis of the CoP nanofibers (CoP NFs), the Co_3_O_4_ nanofibers (Co_3_O_4_ NFs) were phosphorized under the same calcination conditions as CoMoP NFs.

### 2.4. Characterizations

The morphologies of the catalysts were detected via scanning electron microscopy (SEM, SU70, Hitachi, Japan). The lattice structure and substance information were obtained from powder X-ray diffraction (XRD, Rigaku D/max2600), and the nanostructure of the products was observed using transmission electron microscopy (TEM, FEI, Tecnai TF20) measurement. X-ray photoelectron spectroscopy (XPS) tests were performed on a Thermo ESCALAB 25 0XI instrument using monochromatic Al Ka-ADES (ht = 1486.6 eV) as the source.

### 2.5. Electrochemical Measurements

All the OER and HER electrochemical performances were performed on a VMP3 electrochemical workstation with a three-electrode system in 1 M KOH electrolyte. A standard calomel electrode (SCE) and a graphite rod were used as the reference and counter electrode, respectively. To prepare the working electrode, we mixed the nanofibers, the acetylene black, and the polytetrafluoroethylene (PTFE) binder based on an 8:1:1 weight proportion. Then the mixture was printed on carbon paper and dried for 24 h. The linear sweep voltammetry (LSV) curves were recorded using a scan rate of 5 mV s^−1^ with iR-compensation. The calculated potentials are all referred to as the reversible hydrogen electrode (RHE), obeying the relationship of E*_RHE_* = E*_SCE_* + 0.2415 V + 0.059*pH. Electrochemical impedance spectroscopy (EIS) tests were recorded in the frequency range of 0.01 Hz to 10^4^ Hz to test the transfer resistance (R*ct*). The stability of the electrocatalyst was evaluated by successive cyclic voltammetry (CV) and chronopotentiometry (J = 10 mA cm^−2^) tests. Double-layer capacitance (C*_dl_*) measurement roughly measures the electrochemical active surface area (ECSA) through the linear relationship between different scan rates and the current density. Turnover frequency (TOF) was calculated by scanning the surrounding areas of cyclic voltammetry tests in 1 M PBS solution.

## 3. Results and Discussion

**Scheme 1:**
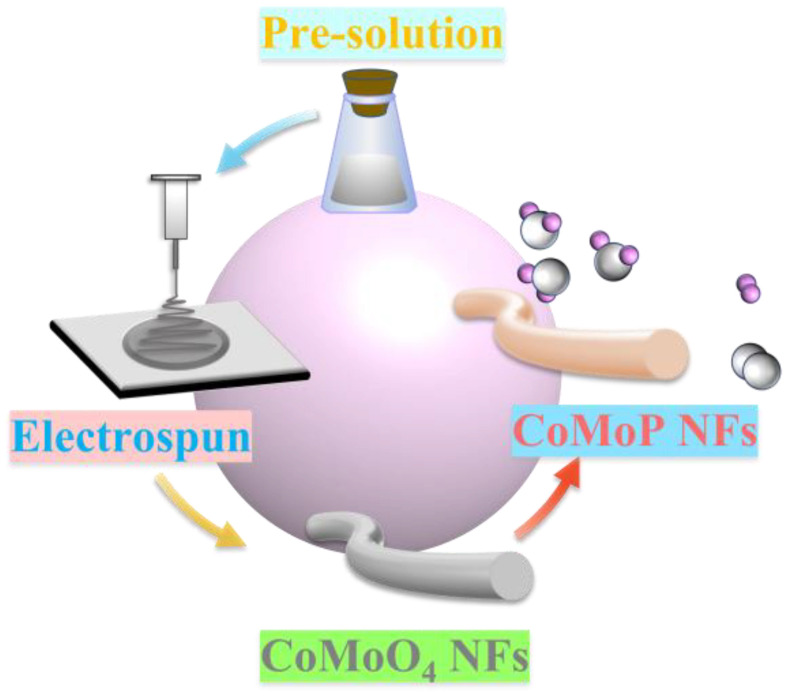
Schematic diagram of the fabrication of the CoMoP NFs.

The facile synthesis strategy is illustrated in Scheme 1; firstly, the one-dimensional CoMoO_4_ NFs precursor was fabricated using electrospinning technology and high-temperature calcination. For the next step, the CoMoP NFs were further produced by the phosphorylation step, with the thermal decomposition NaH_2_PO_2_·H_2_O powder released from the PH_3_ source. The corresponding configuration and composition of the products before and after phosphorylation were thoroughly analyzed by means of scanning electron microscopy (SEM), X-ray diffraction (XRD), transmission electron microscopy (TEM), and X-ray photoelectron spectroscopy (XPS).

**Figure 1:**
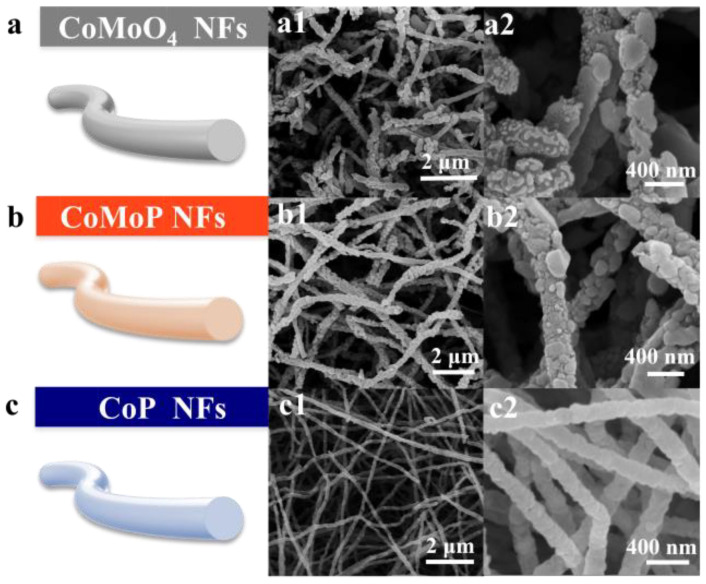
(**a**–**c**) Schematic and SEM images of (**a1**,**a2**) CoMoO_4_ NFs, (**b1**,**b2**) CoMoP NFs, and (**c1**,**c2**) CoP NFs.

**Figure 2:**
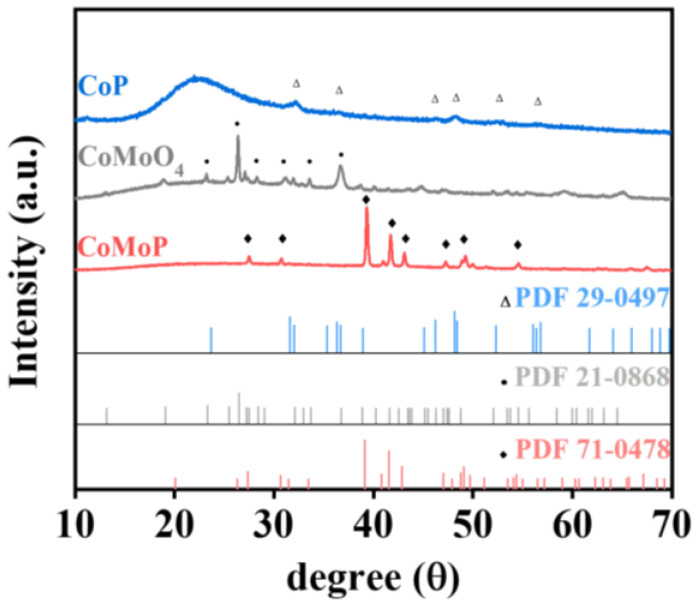
XRD patterns of CoMoO_4_, CoMoP, and CoP NFs.

**Figure 3:**
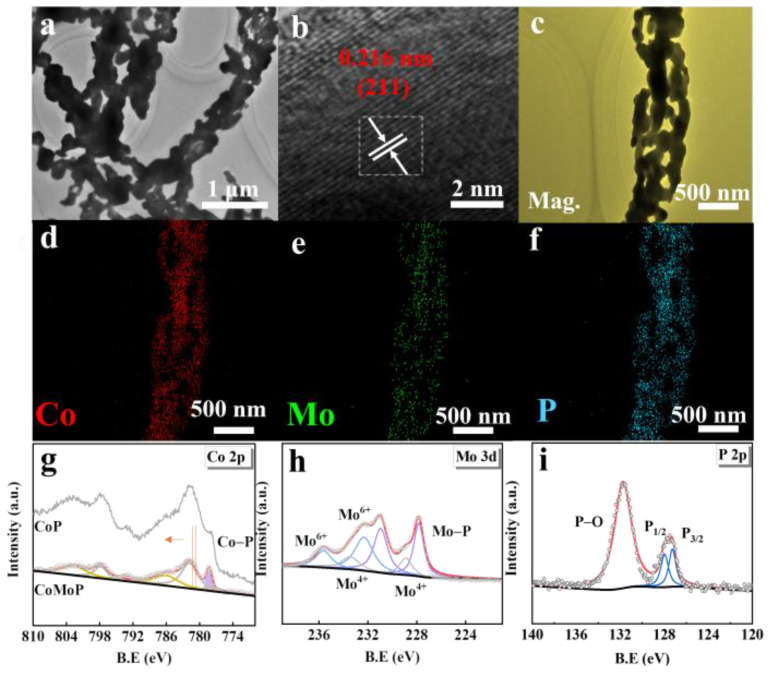
(**a**) TEM image of CoMoP NFs. (**b**) HR-TEM lattice image of CoMoP NFs. (**c**) Magnified TEM image of CoMoP NFs. (**d**–**f**) corresponding element mappings of CoMoP NFs. (**g**) XPS Co 2p spectra of CoMoP NFs and CoP NFs. (**h**,**i**) XPS spectra of Mo 3d and P 2p of CoMoP NFs.

The scanning electron microscopy image shows that the CoMoO_4_ NFs form a dense nanofiber architecture, constituting plenty of uniform small grains, resulting from excluding the PAN template to maintain more desirable voids (Figure 1a). Although the samples experienced a high-temperature gas-solid reaction, as Figure 1b proves, the CoMoP NFs still reserve the initial fiber morphology, with around 500 nm diameter grains which are evenly dispersed in the fibers as well. This stable structure of the CoMoP NFs is nearly unchanged; nevertheless, the outer grains of the fibers become rough, with many visible voids, which facilitates enhanced contact between the charge and the electrolyte surface. In contrast, the tiny size of CoP NFs shows A relatively smooth surface, which was also caught with the SEM imaging for comparison (Figure 1c). Figure 2 shows the XRD pattern of the diffraction peaks of CoMoO_4_ NFs can be indexed into planes of CoMoO_4_ (PDF #21-0868). In the subsequent chemical phosphorylation step, all the diffraction peaks of the resulting materials (heated at 800 °C) can be well indexed within the CoMoP (PDF #71-0478) phase [35]. Additionally, the XRD result proves that the CoP NFs were successfully synthesized. To further verify the morphology of the obtained CoMoP NFs, the TEM images can reveal more details. The thickness of the CoMoP NFs is almost 500 nm, which presents a loose fiber structure loaded with pores (after the phosphorylation) due to the grains expanding in high-heat cavitation (Figure 3a). As is shown in Figure 3b, there are some distinct crystal lattices in the darker areas, corresponding with the selected areas. The measure of the lattice stripe width is 0.216 nm, corresponding to the (211) crystal plane of the CoMoP NFs, which characterizes the XRD result. At the same time, in Figure 3c–f, the magnified images also show the details of the CoMoP NFs, whereby some of the element mapping diagrams can be seen, showing that the Co, Mo, and P elements are distributed evenly within the fibers. Furthermore, the energy dispersive X-ray detector (EDX) characterizations are shown in Figure S1. The TEM images and element mappings of the CoMoO_4_ precursor were obtained, which demonstrates that a lattice stripe size of 0.332 nm (in the HR-TEM lattice image) matches the (002) phase of XRD spectra. The Co, Mo, and O elements are uniformly distributed over the CoMoO_4_ NFs (Figure S2).

In order to further research the electron structures and element valences of the CoMoP NFs, X-ray photoelectron spectroscopy (XPS) depth profiling was carried out. The XPS spectra indicate the distribution of the Co, Mo, and P elements in the CoMoP NFs; the surface distributed Co, Mo, and P signals were observed on the nanofibers. In the high-resolution Co 2p spectra (Figure 3g), two peaks, located at 783.80 eV and 800.43 eV, are allocated to shakeup satellites. The peak belongs to Co 2p_3/2_, for which the position at 781.8 eV corresponds to the typical Co oxide species on the surface. The strong Co-P peak located at 778.25 eV proves that a significant quantity of metal phosphates exists on the surface of the CoMoP NFs. In addition, the remaining peaks of Co 2p_1/2_, which are located at 793.12 and 797.8 eV, endorsed the binding energy of the oxide species and Co-P, respectively. Compared to the Co 2p spectra of the pure CoP NFs, the binding energy has up-shifted by ~0.4 eV, which suggests that transfers of electronic and structure modulation exist within the inducing Mo element [36,37,38]. For the Mo 3d spectrum of the sample, the peaks at 227.83 and 230.88 eV endorse Mo-P bonding, and the peaks at 228.86 and 233.6 eV are matched to the Mo^4+^ species, with the other peaks located at 232.20 and 235.64 eV assigned to Mo^6+^ species (Figure 3h). These multivalent molybdenum species are oxides formed due to the exposure of the sample surface to air. As the P 2p spectra show (Figure 3i), the distinct peaks at 129.46 and 130.16 eV correspond to M-P (M, such as Co, Mo) of the bimetal phosphides, implying the presence of many negative ions, which benefit the capturing of protons toward water-splitting. The binding energy at 133.94 eV is a unique peak, owing to the formation of P-O species on the sample from surface oxidation [39,40].

**Figure 4:**
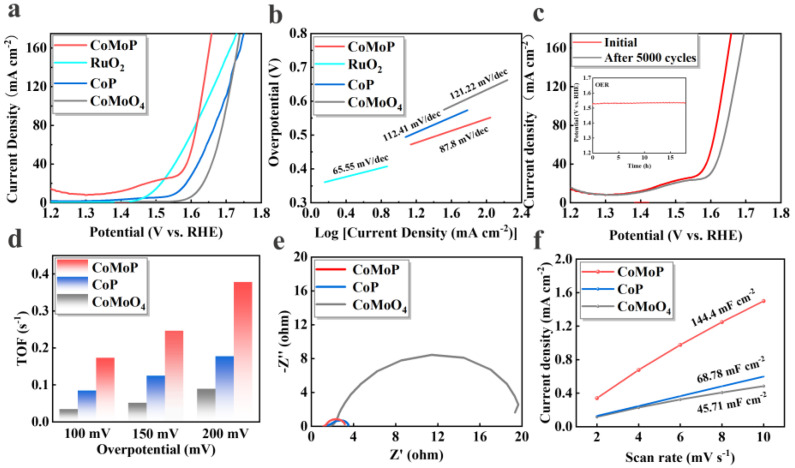
(**a**) LSV curves of RuO_2_, CoMoP, CoP, and CoMoO_4_ NFs. (**b**) Corresponding OER Tafel plots. (**c**) The stability of the CoMoP NFs for 5000 CV cycles and chronopotentiometry testing at a current density of 10 mA cm^−2^. (**d**) Comparison of TOF values under different overpotentials for CoMoP, CoP, and CoMoO_4_ NFs. (**e**) EIS pattern of the above catalysts. (**f**) The plots of the current density versus the scan rate of the above electrocatalysts.

The electrochemical performance of the CoMoP NFs toward the OER reaction was studied in 1M KOH, with a standard three-electrode system. To distinctly find the activity of the CoMoP NFs, along with the CoMoO_4_ NFs and the CoP NFs were set against a comparison sample, which was also measured with the same test system. In order to be able to visually evaluate the OER catalytic performance of each catalyst, the overpotentials under the same current density were compared. In Figure 4a, RuO_2_ shows the lowest overpotential, with a current density of 50 mA cm^−2^, for which the CoMoP NFs were relatively closer than RuO_2_, with an overpotential of 362 mV under the same current density. The CoMoP NFs exhibit good characteristics, better than that of the CoP NFs (403 mV) and CoMoO_4_ NFs (438 mV). More interesting, a low overpotential of enduring a high current density (of 100 mA cm^−2^) only requires 391 mV, which is still lower than that of the CoP NFs (459 mV) and CoMoO_4_ NFs (470 mV) (Figure S3). The synergistic effects of the bimetallic phosphides can improve the catalytic quality for oxygen production, even during a high current density, which, combined with the stable fiber-structure advantages, may find wide application in the field of high-density energy catalysis [41].

At the same time, we calculated the corresponding slope of the Tafel curves (converted from the polarization curves), which were used to represent different catalysts’ kinetic performance during electrochemical reactions. As is shown in Figure 4b, the CoMoP NFs exhibit a smaller Tafel slope of 87.8 mV dec^−1^, which is not only similar to the Tafel value of RuO_2_, but is also better than the precatalyst and pure CoP NFs (112.41 mV dec^−1^ and 121.22 mV dec^−1^), which means the reaction converts the different intermediates, such as *OOH and *OH, quicker for the production of oxygen. CoP NFs perform worse than the CoMoP NFs for OER catalysis, which indicates that combining the heterogeneous atom, Mo, could induce more favorable electrocatalytic kinetics for CoMoP NFs. When comparing electrochemical capability, long-term stability was considered as the basilic factor for the catalyst. As the results show, the final cycle curve for the CoMoP NFs still has a low potential, with a current density of 50 mA cm^−2^, while chronopotentiometry testing revealed that the CoMoP NFs can keep stable for more hours (Figure 4c). This may be due to the one-dimensional nanostructure of the catalyst facilitating the diffusion of the charge and ions on the surface, allowing the catalysts and the electrolyte solution to make contact abundantly, thereby fixing its OER activity during long-term tests.

In order to analyze the excellent intrinsic activity of CoMoP nanofibers, The essential properties of the catalysts were tested by comparing the TOF, which can also reveal the reaction rate in units of time. At an overpotential of 100 mV, a value of 0.173 s^−1^ was achieved by the CoMoP NFs, higher than that of the CoP NFs (0.084 s^−1^) and CoMoO_4_ NFs (0.034 s^−1^). The CoMoP NFs also delivered the best TOF values (0.246 s^−1^, 0.378 s^−1^) for overpotentials of 150 and 200 mV (Figure 4d). The corresponding measured CV curves of the above samples in 1 M PBS solution are illustrated in Figure S4. The charge transfer kinetics during OER were further explored by EIS measurements. It is obviously shown that the charge transfer resistance of the CoMoP NFs is the smallest (2.391 Ω), better than that of the CoMoO_4_ NFs and CoP NFs (18.7 and 2.903 Ω), which demonstrates a faster electron transfer act on the CoMoP catalyst (Figure 4e). The ECSA values of the different products were calculated by measuring the relationship between (C*_dl_*) and the specific capacitance. CV curves at different scan rates (from 2–10 mV s^−1^) were performed over a small voltage range. The smallest C*_dl_* value of 144.4 mF cm^−2^ for the CoMoP NFs was higher than the C*_dl_* value obtained from the CoP NFs and CoMoO_4_ NFs, respectively (68.78 and 45.71 mF cm^−2^). As was expected, the CoMoP NFs exhibit a larger C*_dl_* value due to having more active sites available for the reaction, indicating a higher proportion of electrochemically active area available to the CoMoP NFs (Figure 4f). The measured CV patterns of the catalysts are shown in Figure S5. The extra desirable results of the CoMoP NFs further proves their superiority to other catalysts for producing oxygen in basic feature tests.

**Figure 5:**
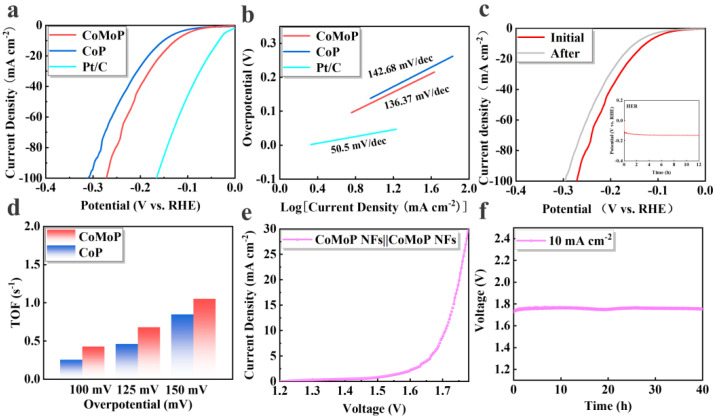
(**a**) LSV curves of Pt/C, CoMoP and CoP NFs. (**b**) Corresponding HER Tafel plots. (**c**) The long-term stability of CoMoP nanofibers for 4000 CV cycles and chronopotentiometry tests at a current density of 10 mA cm^−2^. (**d**) Corresponding TOF values of CoMoP and CoP NFs. (**e**) LSV curves for CoMoP NFs||CoMoP NFs electrolyzers in 1 M KOH in a two-electrode configuration. (**f**) The curves for the chronopotentiometry tests for CoMoP NFs||CoMoP NFs at a current density of 10 mA cm^−2^.

Detecting catalytic behavior on the other side is equally important in water splitting; The HER performance of Pt/C, CoMoP NFs, and CoP NFs were also determined in 1M KOH using a typical three-electrode system. Figure 5a shows the absolute advantage of the Pt/C catalyst for HER. The CoMoP NFs demonstrate better catalytic ability than the CoP NFs, while the LSV curves clearly show that the CoMoP NFs offer the lower overpotential (126 mV), holding a current density of 10 mA cm^−2^, while the CoP NFs need a higher overpotential of 140 mV. The Tafel slope of the CoMoP NFs was computed to be 136.37 mV dec^−1^, which was better than that of the CoP NFs (142.68 mV dec^−1^) (Figure 5b). The low Tafel slope means that the coupling of the different metal elements to the P atom and the suitable structure indeed improves HER activity. Stability is also a pivotal performance aspect of a catalyst. As is shown in Figure 5c, the hydrogen evolution overpotential showed negligible variation after 4000 CV cycles for the stability test (for an overpotential of 142 mV at a current density of 10 mA cm^−2^). Additionally, the catalyst can maintain a potential over 10 h under a constant current density for the chronopotentiometry test, which demonstrated that the CoMoP NFs are stable in HER tests. In order to detect the pure electrochemical activity, we obtained the TOF values of the CoMoP NFs (0.424 s^−1^) and CoP NFs (0.25 s^−1^) for an overpotential 100 mV; the CoMoP NFs also showed higher values at overpotentials of 125 mV and 200 mV (0.676 s^−1^,1.049 s^−1^), which are shown in Figure 5d. This is attributed to excellent stability and good conductivity, which reveals that the CoMoP NFs represent a promising catalyst in the production of hydrogen [42].

Based on their super advanced performance, both toward the production of oxygen and hydrogen in 1 M KOH, we further tested the overall water-splitting ability of the CoMoP NFs for use as both electrodes in a typical system. Herein, the electrochemical test results for water splitting are shown in Figure 5e. The LSV curve shows that the catalyst can drive a 10 mA cm^−2^ current density at a cell voltage of only 1.75 V, which demonstrates that the CoMoP NFs can act as an excellent bifunctional catalyst. As was expected, in Figure 5f, the line barely shifts after 40 h of stability testing using a current density of 10 mA cm^−2^ and the CoMoP NFs for the chronopotentiometry tests, meaning that superior performance of overall water splitting occurred on the surface.

**Figure 6:**
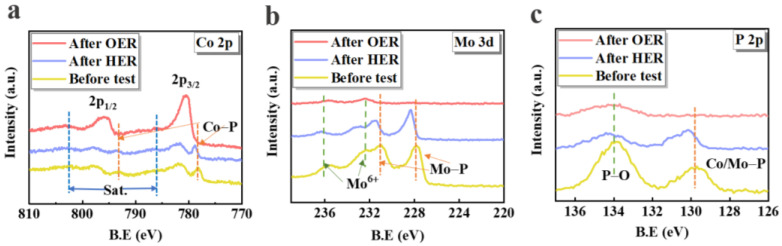
High-resolution XPS spectra of CoMoP NFs-virgin, post HER, and post OER; (**a**) Co 2p, (**b**) Mo 3d, and (**c**) P 2p.

**Figure 7:**
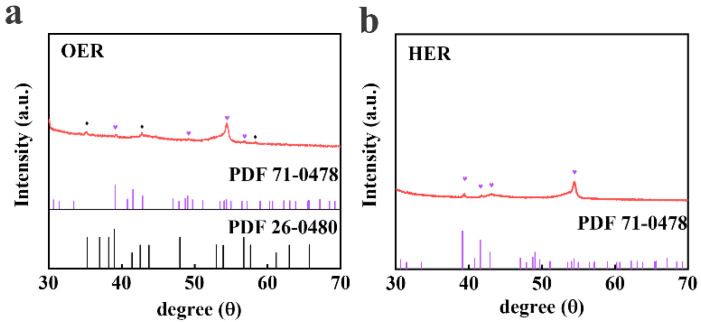
XRD patterns of CoMoP NFs after stability test; (**a**) post OER, and (**b**) post HER.

In order to depth detect the reaction mechanism (related to splendid electrocatalytic activity) and provide the design principles for efficient catalysts, the high-resolution XPS analyses were further measured. After the test, the sample also contains the corresponding elements of Co, Mo, and P. Firstly, concerning the Co 2p core level of OE and the CoMoP NFs after a long-term test, the characteristic peaks for Co−P largely disappear, with high-state species being generated. At the same time, we analyzed other peaks of Co which were strong; these changes, which are due to the element Co on the surface, are mainly caused by electron restructuring. For the CoMoP NFs electrode in the HER test, the high density of the Co 2p peaks showed a little shift when compared with the original chemical state (Figure 6a). The Mo 3d peaks shift toward higher binding energy, which means more high-state Mo species were generated in the KOH electrolyte, and Mo-P peaks also disappeared after the OER test, while the Mo peaks maintained stable chemical states in the HER test (Figure 6b). Additionally, the M-P peaks in the P 2p XPS pattern of the as-tested CoMoP NFs in oxygen evolution are disappearing; in contrast, the P 2p_3/2_ and P 2p_1/2_ pair in the P 2p spectrum remained stable in the HER test (Figure 6c). As Figure 7 shows, the two sides of the reaction still exist for the CoMoP, which reveals the stability of the catalyst. More interestingly, the CoOOH species (PDF 26-0480) are synthesized in Co-active sites during the OER test; meanwhile, during the HER tests, there was no apparent change in the samples. Such results indicate the suitable structural robustness of the binary TMP catalysts and the coupling synergy between the different metal elements enhanced the instinctive activity of the catalyst. To sum up, these findings show the excellent performance of the CoMoP NFs, proving the huge potential for binary TMPs as bifunctional electrochemical catalysts [43,44].

## 4. Conclusions

By synthesizing a 1D binary phosphides catalyst (CoMoP NFs) using a simple electrospinning method and gas-solid reactions, we explored the availability of electrocatalytic activity in an alkaline environment. For the OER process, the CoMoP NFs reached a current density of 100 mA cm^−2^ while only requiring a low overpotential of 390 mV, which was superior to the results of the CoP NFs and CoMoO_4_ NFs and kept the best performance after 3000 cycles. During the HER test, the CoMoP NFs only required 132 mV for the production of hydrogen using a current density of 10 mA cm^−2^. After a long period of stability and more CV cycle measurements, the CoMoP NFs still maintained excellent performance after testing. Based on the above characterizations, our results strongly confirm that CoMoP NFs are considerably promoted during the processes of OER and HER, with excellent capacity. Furthermore, the CoMoP NFs only required a low overpotential to deliver a current density of 10 mA cm^−2^ for overall water splitting of up to 40 h. As practical evidence, the bifunctional catalytic potential of CoMoP NFs is promising for offering an easy approach to enhancing the efficiency of electrochemical reactions.

## Data Availability

All the relevant data are included in this published article.

## Supplementary Materials

The following supporting information can be downloaded at: https://www.mdpi.com/article/10.3390/nano12213886/s1, Figure S1. Energy dispersive X-ray spectroscopy of CoMoP NFs; Figure S2. (a,b) TEM image and HR-TEM lattice image of CoMoO_4_ NFs, and (c–e) corresponding element mappings of CoMoO_4_ NFs; Figure S3. Overpotentials of CoMoP NFs, CoP NFs, and CoMoO_4_ NFs at current densities of 50 mA cm^−2^ and 100 mA cm^−2^ for OER test; Figure S4 Cyclic voltammograms of (a) CoMoP NFs, (b) CoP NFs, and (c) CoMoO_4_ NFs with a potential range from −0.2–0.6 V (V vs. RHE) in 1M PBS solution; Figure S5. Cyclic voltammograms of (a) CoMoP NFs, (b) CoP NFs, and (c) CoMoO_4_ NFs in a potential range from 0.2–0.3 V (V vs. SCE) in 1 M KOH.
