# Supplementary material for: One-Dimensional CoMoP Nanostructures as Bifunctional Electrodes for Overall Water Splitting
Source: Nanomaterials (Basel). 2022 Nov 3;12(21):3886. doi: 10.3390/nano12213886 (PMC9658805; doi:10.3390/nano12213886)
Supplement: Supplementary file 1 [file nanomaterials-12-03886-s001.zip › nanomaterials-1977245-supplementary.pdf]

nanomaterials

Supplementary Material

One-Dimensional CoMoP Nanostructures as Bifunctional Electrodes for Overall Water Splitting

Xin Chang [1], Jun Yan [1], Xinyao Ding [1], Yaozu Jia [1], Shijie Li [2,*] and Mingyi Zhang [1,*]

[1] Key Laboratory for Photonic and Electronic Bandgap Materials, Ministry of Education, School of Physics and Electronic Engineering, Harbin Normal University, Harbin 150025, China
[2] National Engineering Research Center for Marine Aquaculture, Institute of Innovation & Application, Zhejiang Ocean University, Zhoushan 316022, China
* Correspondence: lishijie@zjou.edu.cn (S.L.); zhangmingyi@hrbnu.edu.cn (M.Z.)

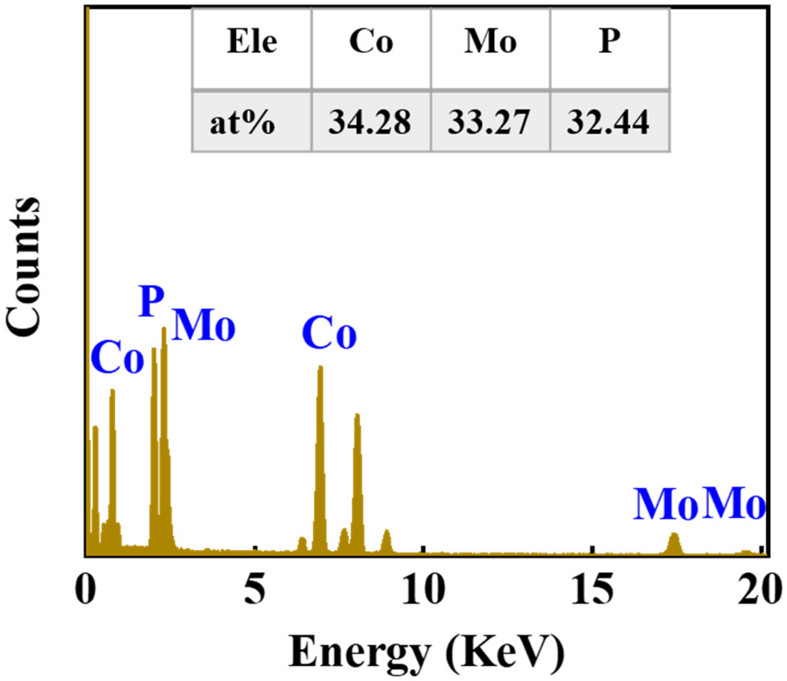

Figure S1. Energy dispersive X-ray spectroscopy of CoMoP NFs.

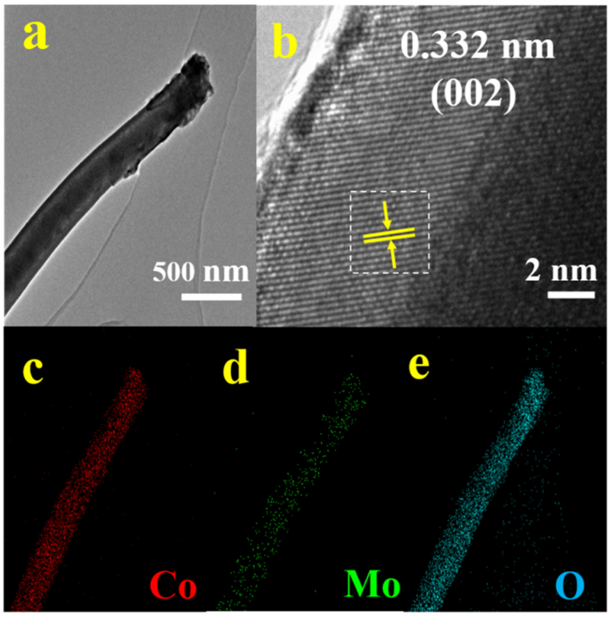

Figure S2. (a,b) TEM image and HR-TEM lattice image of $CoMoO_4$ NFs, and (c-e) corresponding element mappings of $CoMoO_4$ NFs.

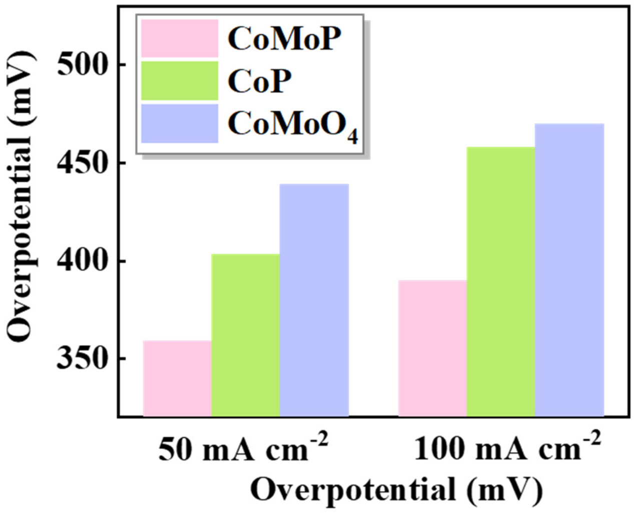

Figure S3. Overpotentials of CoMoP NFs, CoP NFs, and $CoMoO_4$ NFs at current densities of 50 mA cm^{-2} and 100 mA cm^{-2} for OER test.

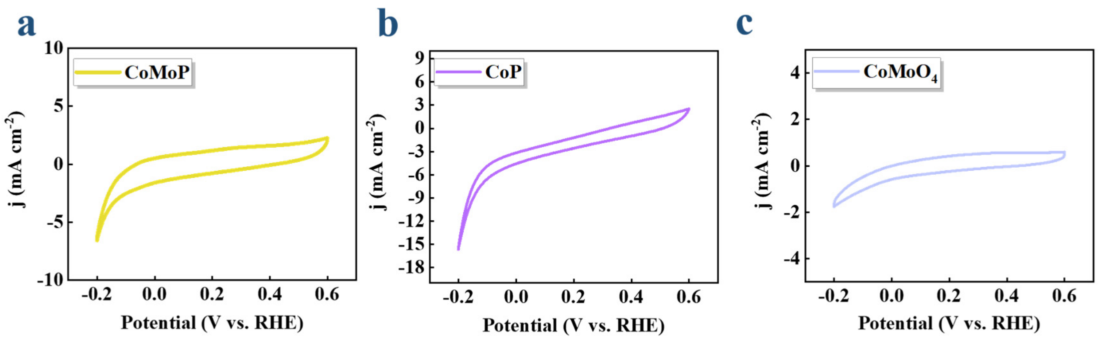

Figure S4 Cyclic voltammograms of (a) CoMoP NFs, (b) CoP NFs, and (c) $CoMoO_4$ NFs with a potential range from -0.2-0.6 V (V vs. RHE) in 1M PBS solution.

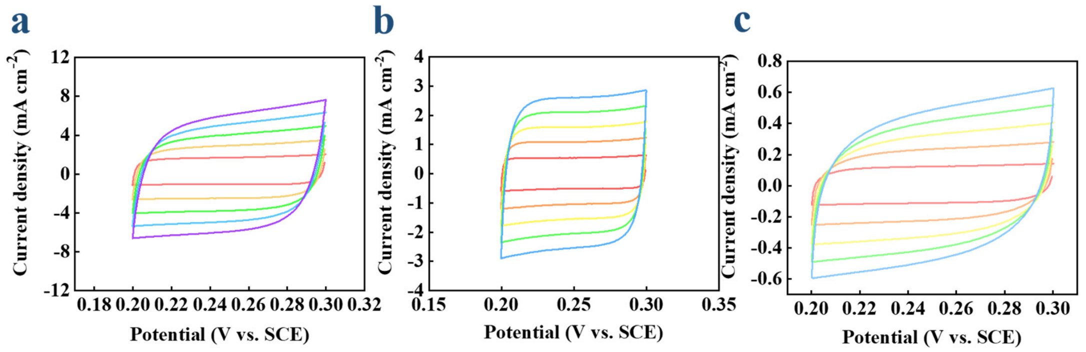

Figure S5. Cyclic voltammograms of (a) CoMoP NFs, (b) CoP NFs, and (c) $CoMoO_4$ NFs in a potential range from 0.2-0.3 V (V vs. SCE) in 1 M KOH.
